# Experimental Study on Mechanical Properties and Porosity of Organic Microcapsules Based Self-Healing Cementitious Composite

**DOI:** 10.3390/ma10010020

**Published:** 2017-01-01

**Authors:** Xianfeng Wang, Peipei Sun, Ningxu Han, Feng Xing

**Affiliations:** Guangdong Provincial Key Laboratory of Durability for Marine Civil Engineering, College of Civil Engineering, Shenzhen University, Shenzhen 518060, Guangdong, China; xfw@szu.edu.cn (X.W.); sunpeipei@agile.com.cn (P.S.); nxhan@szu.edu.cn (N.H.)

**Keywords:** microcapsules, healing rate, recovery rate, pore size, dynamic modulus

## Abstract

Encapsulation of healing agents embedded in a material matrix has become one of the major approaches for achieving self-healing function in cementitious materials in recent years. A novel type of microcapsules based self-healing cementitious composite was developed in Guangdong Provincial Key Laboratory of Durability for Marine Civil Engineering, Shenzhen University. In this study, both macro performance and the microstructure of the composite are investigated. The macro performance was evaluated by employing the compressive strength and the dynamic modulus, whereas the microstructure was represented by the pore structure parameters such as porosity, cumulative-pore volume, and average-pore diameter, which are significantly correlated to the pore-size distribution and the compressive strength. The results showed that both the compressive strength and the dynamic modulus, as well as the pore structure parameters such as porosity, cumulative-pore volume, and average-pore diameter of the specimen decrease to some extent with the amount of microcapsules. However, the self-healing rate and the recovery rate of the specimen performance and the pore-structure parameters increase with the amount of microcapsules. The results should confirm the self-healing function of microcapsules in the cementitious composite from macroscopic and microscopic viewpoints.

## 1. Introduction

Concrete has been one of the most widely used building materials in the world owing to its low energy consumption, low cost, and relatively high durability [1]. However, in the natural environment, there is a risk of erosion. Materials’ age and environmental effects result in concrete microcracks, local damage, and fracture. In particular, in actual concrete structures, micro-cracks are difficult to detect accurately because of the limitations of the detection technology; moreover, the conventional method cannot effectively repair the internal structure of these invisible microcracks. If these microcracks are not effectively repaired, it will affect the normal performance and service life of the structure, and may lead to macroscopic cracking and cause structural brittle fracture, and even lead to a catastrophic accident [2]. Hence, it is necessary to develop new repairing techniques and materials that are able to perceive material damage, and passively and automatically repair the damaged site, thereby restoring the mechanical properties and durability of concrete.

There are several self-healing approaches, such as microbiology, shape memory alloy or polymer, extending hydration of cementitious admixtures, and microencapsulation technology, which are possible because the material has a core-shell tiny container structure. Xing et al. [3] developed a self-healing technique using organic microcapsules for cement paste. In their study, the integrity of organic microcapsules was maintained while preparing the cement paste, and the microcapsules ruptured when the cracks passed through them. An element analysis provided definite proof of the healing phenomenon on the crack faces.

Microcapsules, as temporary vessels, hold the healing agent until damage induced trigger occurs. The repair principle of microcapsules based on the self-healing cementitious composites is similar to the principle of bionics, wherein a crack is initiated and then propagated. The embedded microcapsules ruptured under stresses, and subsequently, the healing agent was released into the crack plane through the capillary action to achieve the healing function, thereby inhibiting crack propagation and repairing the crack, even restoring or improving the effect of the material strength [4,5,6]. It is efficient and has unique advantages from the viewpoint of durability. This method can heal cracks induced at places difficult to access; moreover, the method is relatively low cost [7]. Although the method needs to be further developed and investigated in the future, the encapsulation scheme seems to be a promising approach for self-healing [8,9].

In recent years, a considerable number of studies have been conducted following this route. Su et al. [10,11,12] used a type of rejuvenator as the core materials to investigate the mechanical healing behaviors of bitumen using a modified beam on elastic foundation method, wherein three types of microcapsules with different mean sizes and shell thicknesses were considered. Dong et al. [13,14] investigated the self-healing capacity of a cementitious composite containing organic microcapsules by evaluating the crack healing effect, mechanical property, as well as chloride permeability. Their experimental results revealed that the crack-healing ratios were 20%–45%, and the healing ratios of the compressive strength and impermeability were approximately 13% and 19.8%, respectively. They [15] also developed a chemical self-healing system, for which experiments were conducted in a stimulated concrete pore solution. The smart release behavior of the healing agent in the microcapsule, characterized by the ethylene diamine tetra-acetic acid titration method, was a function of time, and controlled by the wall thickness of the microcapsule. Lv et al. [16] developed a type of polymeric microcapsule with phenol–formaldehyde resin for the shell and dicyclopentadiene as the healing agent for the self-healing of microcracks in cementitious materials. The chemical stability of the microcapsules and the trigger performance were verified in a simulated concrete pore solution and hardened cement paste specimens.

De Belie’s group [17,18] studied the self-healing concrete by employing the microencapsulated bacterial spores, wherein the breakage of the microcapsules upon cracking was verified using scanning electron microscope (SEM), and the self-healing capacity was evaluated via the crack healing ratio and the water permeability. Their results showed that the healing ratios in the specimens were 48%–80%. They also studied the microstructure of the capsules containing self-healing materials by using micro-computed tomography. The three-dimensional distribution and de-bonding of the microcapsules in their native state in a polymer system with self-healing properties were indicated.

In addition, there are a number of review papers [19,20,21,22,23,24,25] in the field of self-healing materials, and five international conferences were conducted on self-healing materials [26,27]. Souradeep and Kua [24] suggested eight factors that affect the effectiveness of self-healing by encapsulation, which included the following: (1) robustness during mixing and (2) probability of cracks encountering the capsules. They indicated that there is a lack of research on the efficacy of self-healing in an actual application environment. As some fundamental issues, such as the control of fabrication, have not been clarified, most research is still in the laboratory level. Muhammad et al. [25] reviewed the self-healing measurement methods, particularly concerning the healing effect of the width, depth, and length of cracks. They indicated that few studies on the healing efficiency were conducted at the microstructure or nanostructure level. It is also found that, for microcapsules based self-healing materials, there are still fewer systematic studies on the healing behavior from the viewpoints of porosity and the correlation between the macro behavior and microstructure.

Moreover, with the hardening of the concrete structure, the evaporation of free water inside the concrete may generate pores, and the presence of pores of different sizes is an important component of hardened concrete structure, which is an important factor influencing its performance. Mercury intrusion porosimetry (MIP) [28,29,30] is commonly used to evaluate the pore structure of cementitious materials. By using MIP, the pore-size distribution may be determined mainly based on the relationship between the amount of mercury flowing into the porous system of the concrete materials and the applied pressure. It is capable of measuring a vast range of pore entry radii varying from 6 nm to 400 μm.

In this study, the organic microcapsules were prepared and used to make the self-healing cementitious composite specimens. This type of microencapsulation approach is based on the physical trigger. The organic microcapsules with epoxy core can produce ductility for the cementitious composites and produce relatively high healing rate with the amount of microcapsules. This is the advantage. However, embedding the microcapsules may weaken the initial strength of the specimen; hence, an optimum dosage should be determined before industrial application. The healing efficiency of the specimens was investigated based on the strength, dynamic modulus, as well as the pore-size distribution via MIP test. The damage was inflicted to the specimens by applying uniform compression. The pore-size distributions at intact, damaged, and healed states were measured, and the healing ratios, as well as the recovery ratios were determined. Then, the pore parameters, such as porosity, cumulative-pore volume, and average-pore diameter, were obtained, and further research on the pore-size distribution model of mortars was conducted, from which the healing effect of the microcapsules present in the cementitious materials was validated.

## 2. Experimental Scheme

### 2.1. Materials and Specimens

#### 2.1.1. Preparation of Microcapsules

The organic microcapsules were synthesized at Guangdong Provincial Key Laboratory of Durability for Marine Civil Engineering, Shenzhen University. The shell material is urea formoldehyde (UF), and the core-healing agent is epoxy. The materials used for the synthesis are urea (analytical reagent: AR) obtained from Tianjin Jinfeng Chemical Ltd. Co. (Tianjin, China), formaldehyde solution (AR) obtained from Tianjin Baishi Chemical Ltd. Co. (Tianjin, China), triethanolamine (AR) obtained from Tianjin Fuyu Chemical Ltd. Co. (Tianjin, China), butyl glycidyl ether (BGE) obtained from Shanghai Bangcheng Chemical Ltd. Co. (Tianjin, China), a type of epoxy resin E-51 obained from Shenzhen Yoshida Chemical Ltd. Co. (Shenzhen, China), and sulfuric acid obtained from Tianjin Guangfu Institute of Fine Chemicals. The details of the synthesis can be found in a previous study [6].

**Figure 1:**
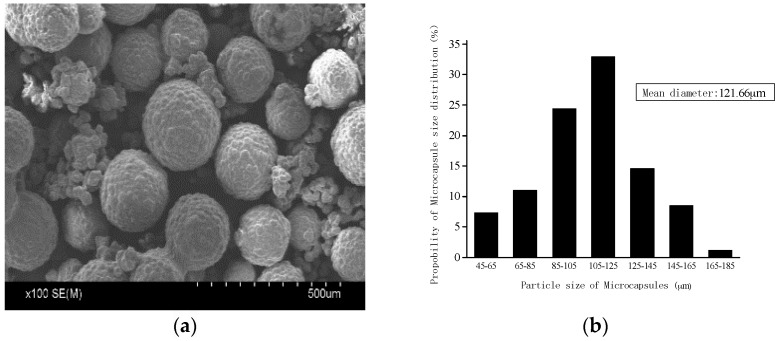
Organic microcapsules: (**a**) SEM images of the microcapsules; (**b**) Particle-size distribution of the microcapsules.

Table 1 lists the detailed parameters of the microcapsules. Figure 1a shows an image of the microcapsules analyzed using a SEM. Figure 1b shows the particle-size distribution of the microcapsules, wherein the mean diameter is 121.66 μm. The diameter distribution was determined by using an optical microscope and SEM for accounting the samples of 300 microcapsules. The shell thickness of the organic microcapsules was tested using SEM images when the microcapsules were ruptured by grinding. The capsule core content was obtained by extraction [31]. The procedure was as follows: weighing a certain amount of dried microcapsules, full grinding out the core agent, and then placing the ground material in acetone and placed 3d; during the period, acetone was changed every 24 h so that the core content fully flowed out. Next, the ground material was dried in a drying oven, and the remaining material formed the wall of the microcapsule. The core content *w*_cc_ can be calculated as

(1) $$w_{cc}=\frac{\left(m_{capsule}-m_{shell}\right)}{m_{capsule}}\times 100\%$$

where $m_{capsule}$ is the mass of the measured microcapsules, and $m_{shell}$ is the mass of the microcapsule wall.

#### 2.1.2. Preparation of the Microcapsule-Based Cementitious Materials and Specimens

To build the microcapsule-based self-healing cementitious composite, the following materials were used: China portland Cement GB-175-2007 [32] PII42.5R type from Guangzhou Zhujiang Cement Ltd. Company (Guangzhou, China); drinkable tap water; GB/T17671-1999 ISO standard sand [33] from Xiamen Isiou Ltd. Company (Xiamen, China); a curing agent MC120D from Guangzhou Kawai Electronic Materials Ltd. Company (Guangzhou, China).

The mortar specimens were prepared using the mix proportion, given in Table 2, wherein the water/cement and the binder/sand ratios were 0.5 and 1:3, respectively. The microcapsule size (diameter of 121.66 μm) and the content (0%, 3%, 6%, and 9% to cement mass) were considered. The amount of catalyst MC120D used was half the amount of the organic microcapsules.

**Figure 2:**
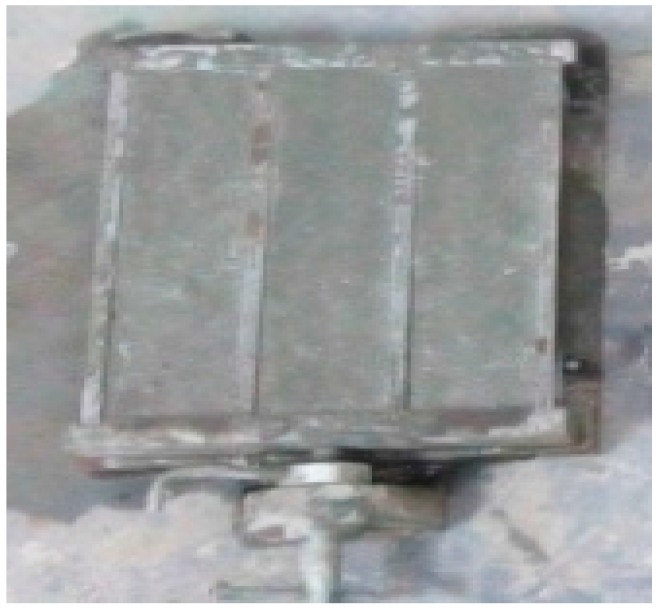
Specimens for test.

**Figure 3:**
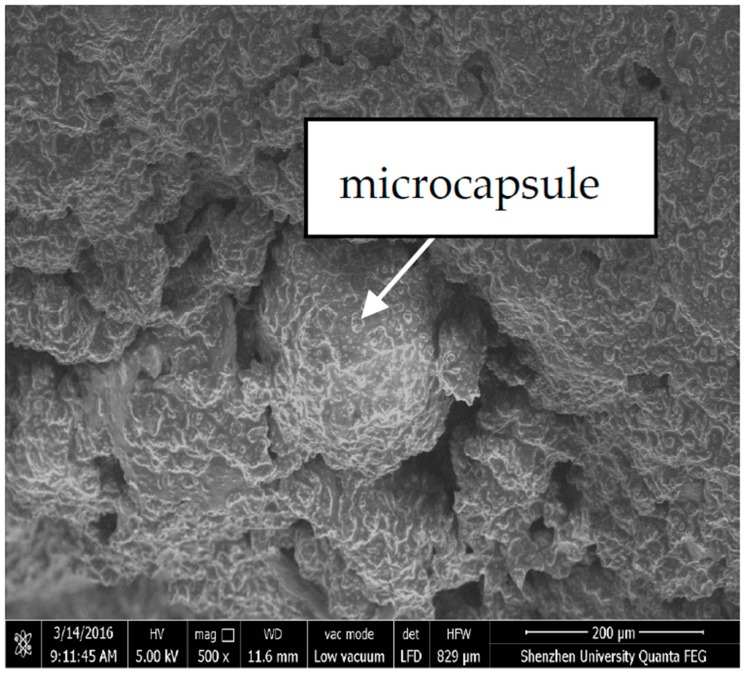
Microcapsules dispersed in the cementitious composite.

The prismatic specimens of dimensions 40 mm × 40 mm × 160 mm were prepared by mixing the microcapsules as well as the catalyst MC120D with water, cement, and sands, as shown in Figure 2. The specimens were demolded after 24 h and cured for 28 days under the same conditions as that of the standard mortar tests (temperature 20 °C, humidity >90%). Figure 3 shows the microcapsules dispersed in the cement mortar observed using SEM.

### 2.2. Experimental Methods

#### 2.2.1. Compressive Strength Test

The compressive strength test was conducted using the testing machine RGM-4010 (REGEL Corp., Shenzhen, China) based on the standard given in a previous study [33]. The loading speed was 2.4 kN/s. The compression strength is the ratio of the maximum compression load to the loading area (40 × 40 mm). Three specimens were grouped as one sample in the test, wherein the average value was used for the representative one.

#### 2.2.2. Preparation of Specimens for Self-Healing Test

Three groups of specimens under the same mix proportion were prepared. The first group was for the compressive strength and pore structure tests at the intact state. The second group was for the test at the damaged state, which was obtained by applying a pre-load of 60% σ_max_ (maximum compressive strength) to the specimens. The third group was for the test at the healed state, which was obtained by curing the wrapped damaged specimens in a curing box below a temperature of 50 °C for 7 days.

The sizes of the specimens for the dynamic mechanical analysis (DMA) test and pore-structure test were much smaller than the standard cement mortar; moreover, they were different for each test. The corresponding specimens were obtained using fine cutting, and were immersed in ethanol for 7 days to terminate hydration. Thereafter, they were placed in a drying box at 60 °C, which were ready for the test. Table 3 gives the test number and the types of the tests types, wherein the sample No. is referred from Table 2.

#### 2.2.3. Dynamic Mechanical Analysis (DMA) Test

The specimens for the DMA test were prepared to dimensions 30 mm × 30 mm × 30 mm. The testing machine was DMA + 1000 from ACOEM Metravib Ltd. Co. (Limonest, France). The frequency was 1 Hz, and the static and the dynamic force were 120 N and 100 N, respectively. The DMA tests were conducted for samples No. 1–4 with a microcapsule size of 121.66 μm. The dynamic moduli were then obtained.

#### 2.2.4. MIP Test

The MIP test has been widely used to test the pore-structure parameters of cement-based materials, such as porosity, critical pore size, threshold pore size, and mean diameter. In this study, Auto Pore 9500 type testing machine from Micromeritics Ltd. Co. (Norcross, GA, USA). was used. The dimensions of the samples were below 10 mm × 10 mm × 10 mm. Both the low and high-pressure analyses were conducted. First, a vacuum condition of less than 40 μm Hg air pressure was obtained. Then, the mercury was pressurized at a pressure range of 0.54 to 29.98 psia. The corresponding pore size ranges were from 6 to 350 μm. After the low-pressure test, the high-pressure test was implemented in a similar manner. The high-pressure range of 36.4–29,906.6 psia corresponded to the measured pore size range of 6.05–4964 nm.

The basic principle of MIP test is to convert the mercury pressure to the pore size by applying the Washburn, as expressed in equation [30].

(2) $$d=\frac{4\gamma \cos\theta }{P}$$

where *d* represents the pore size (μm); γ denotes the mercury surface tension, 0.484 N/m; θ signifies the contact angle of the mercury with the pore wall, 117°; *P* is the mercury pressure of the injected sample (Pa). With the Washburn equation, the pore-size distribution as well as the related pore structure data can be calculated.

## 3. Results and Discussion

### 3.1. Compression Test Results and Discussions

**Figure 4:**
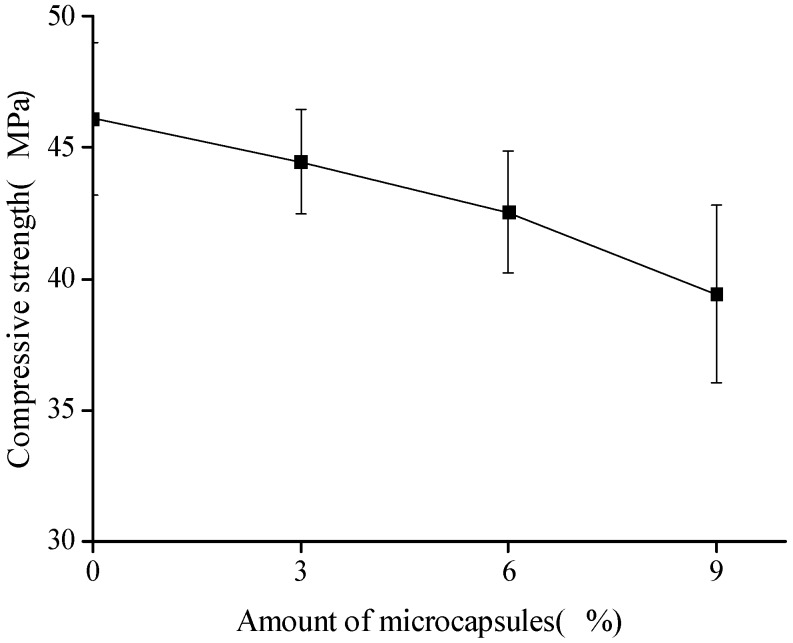
Variation in the compressive strength with the amount of microcapsules.

The compressive strength was obtained by using $\sigma =\frac{F}{b^{2}}$, where *F* is the maximum compressive load and *b* is the lateral length of the specimen (40 mm). Figure 4 shows the variation in the compressive strength with the amount of microcapsules. Herein, for convenience of expression, sample No. 1 was treated as a reference. The compressive strength was 46.1 MPa. The error bars in the figure indicated the standard deviation, wherein the maximum value was 3.4 MPa in the case of 9% microcapsules. The compressive strengths are observed to decrease with the amount of microcapsules. The Young’s modulus of UF is less than 10 GPa, and the Young’s modulus of the cement mortar is in the range of 10–30 GPa. Hence, the lower values of the Young’s modulus and the interface strength make the microcapsules perform as weak phases. The larger the microcapsules, the greater the effect [34]. The compressive strength can be decreased up to 14.5% in the case of 9% microcapsules. However, in the case of small particle sizes, a small amount of microcapsules (3%) may lead to a slight increase (1%) in the strength or not affect the variation. This may be the cause of the filler effect of the microcapsules, which coincide with the results of a previous study [6].

As mentioned previously, the mechanism of the self-healing function achieved by the microcapsules involves the cracking induced rupture of the microcapsules. The core-healing agent flows out and reacts with the catalyst; thereafter, it solidifies and glues to the cracks. To investigate the self-healing efficiency of the mechanical behavior, the recovery rate $\eta_{S-ROC}$ and the healing rate $\eta_{S-HEA}$ are defined, respectively, as

(3) $$\eta_{S-ROC}=\frac{f_{healed}}{f_{original}}\times 100\%\;,$$

(4) $$\eta_{S-HEA}=\frac{f_{healed}-f_{damaged}}{f_{damaged}}\times 100\%\;,$$

where $f_{healed}$ denotes the specimen strength after the healing process (MPa); $f_{original}$ is the original strength of the specimen at 28 days (MPa); $f_{damaged}$ is the strength of the specimen at the damaged state because of the pre-loading (MPa).

**Figure 5:**
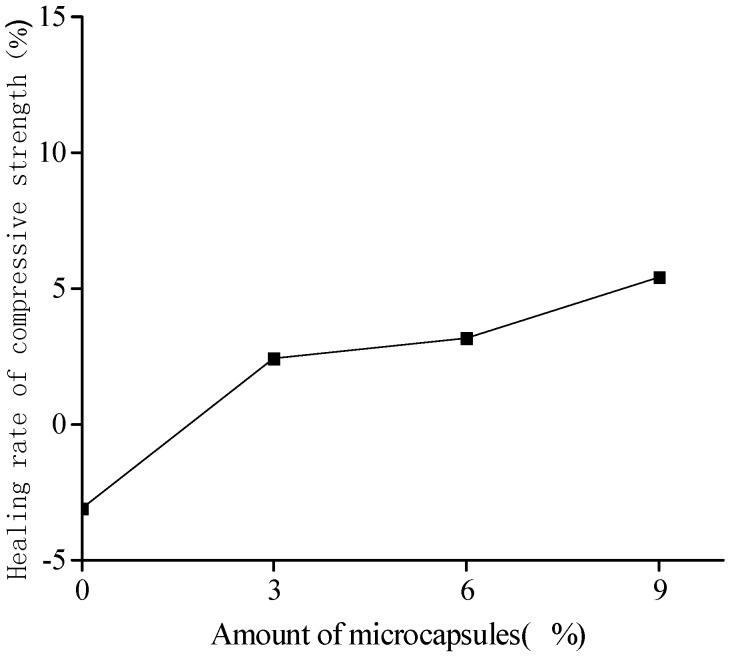
Healing rate (Equation (4)) of compressive strength with amount of microcapsules.

**Figure 6:**
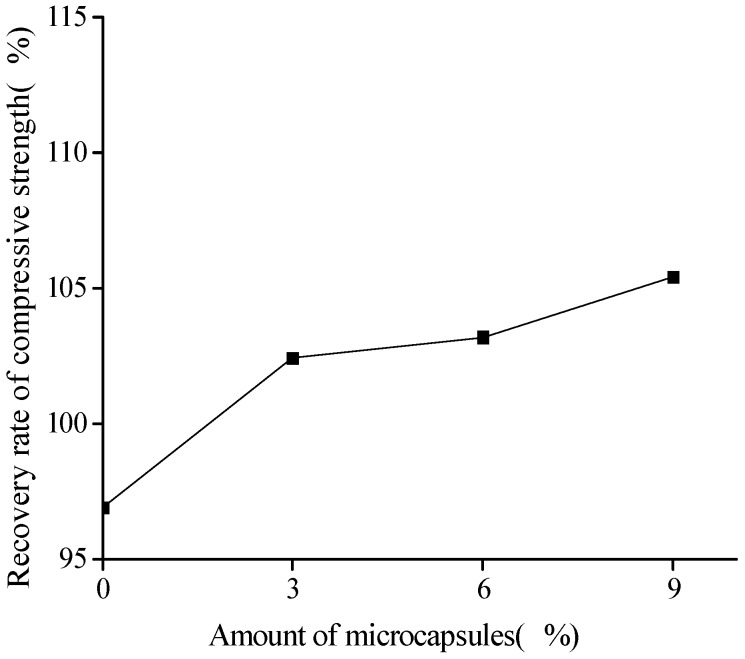
Recovery rate (Equation (3)) of compressive strength with amount of microcapsules.

Figure 5 and Figure 6 show the healing and recovery rates of the compressive strength, respectively. The figures show that, in the absence of microcapsules (reference specimen), the healing and recovery rates of the compressive strength are −3.1% and 96.90%, respectively, which means that, from an overall point of view, the strength is difficult to recovery because of the effect of relatively large cracks, though the negative value arises from the measurement dispersion. The healing and recovery rates are greater than 0% and 100%, respectively, for the specimens containing the microcapsules, which represents a good healing efficiency of the microcapsules. The adhesive reaction glues the cracks and improves the compressive strength. For the cement mortar with the same size, but different content of the microcapsules, which are in the range of 3% to 9%, the healing and recovery rates increased with the increase in the content of microcapsules. The content of microcapsules increases the content of the healing agent, thereby increasing the healing rate. The healing rate of the cement mortars with the microcapsules of content 9% reached 5.42%. This should strongly demonstrate the self-healing effect on the compressive strength.

### 3.2. DMA Results and Discussions

To investigate the self-healing efficiency of the dynamic modulus of the specimen, we use similar definitions of the recovery rate $\eta_{E-ROC}$ and healing rate $\eta_{E-HEA}$ with Equations (3) and (4), respectively, as

(5) $$\eta_{E-ROC}=\frac{E_{healed}}{E_{original}}\times 100\%\;,$$

(6) $$\eta_{E-HEA}=\frac{E_{healed}-E_{damaged}}{E_{damaged}}\times 100\%\;,$$

where $E_{healed}$ denotes the dynamic modulus after the healing process (GPa); $E_{original}$ is the original dynamic modulus of the specimen at 28 days (GPa); $E_{damaged}$ is the dynamic modulus of the specimen at the damaged state (GPa).

**Figure 7:**
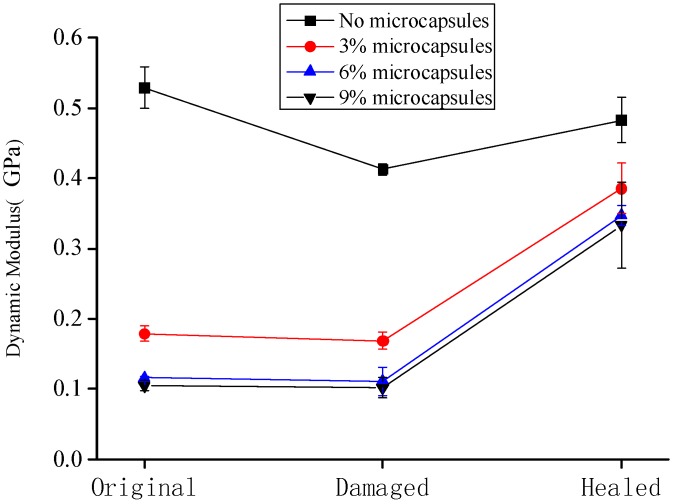
Variation in the dynamic modulus of the specimens.

Figure 7 shows the variation in the dynamic modulus of the specimens at the original, damaged, and healed states. The data are the averaged values of the three specimens. The error bars indicated the standard deviation, wherein the maximum value was 0.061 GPa for 9% of microcapsules after the healing. It is seen that the dynamic modulus of the cement-mortar decreases with the increase in the content of microcapsules. Because of the difference of the elastic modulus between the microcapsules and cement matrix, and the interface between them may also be relatively weak, the stiffness as well as the elastic modulus of the cementitious composite, should be decreased with the content of microcapsules, particularly for the case shown in Figure 7. Moreover, it can be seen that the dynamic modulus of the cementitious composite without microcapsules is considerably higher than that of the composite containing the microcapsules. The dynamic modulus of the specimen after the preloading was lower than that of the original specimens, which indicates that the specimen was damaged after the preloading; the microcracks appeared and the stiffness as well as the elastic modulus of the specimen decreased. The dynamic modulus of the specimen after the healing was higher than that of the damaged sample. In the case of the specimen without microcapsules, the high dynamic modulus could be attributed to the extension of the hydration of the cementitious composite, which self-heals the microcracks. In the case of the specimen containing the microcapsules, the reaction of the healing agent with the catalyst improves the elastic modulus.

**Figure 8:**
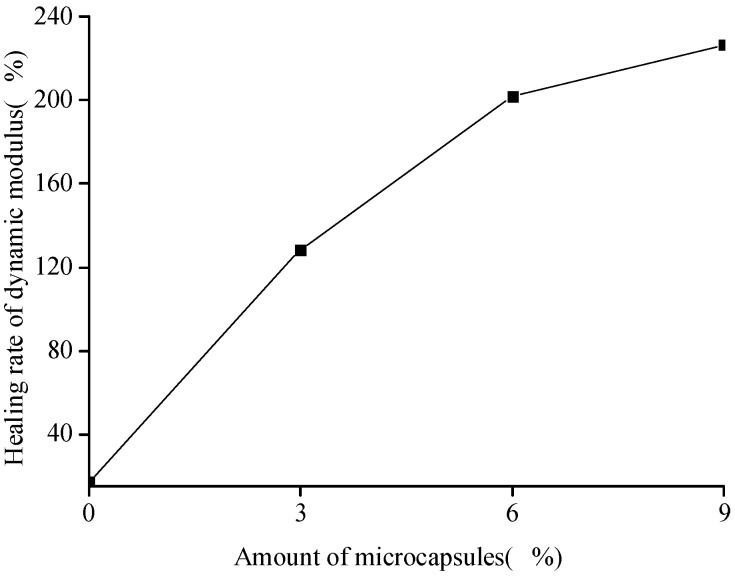
Healing rate (Equation (6)) of the dynamic modulus with the amount of microcapsules.

**Figure 9:**
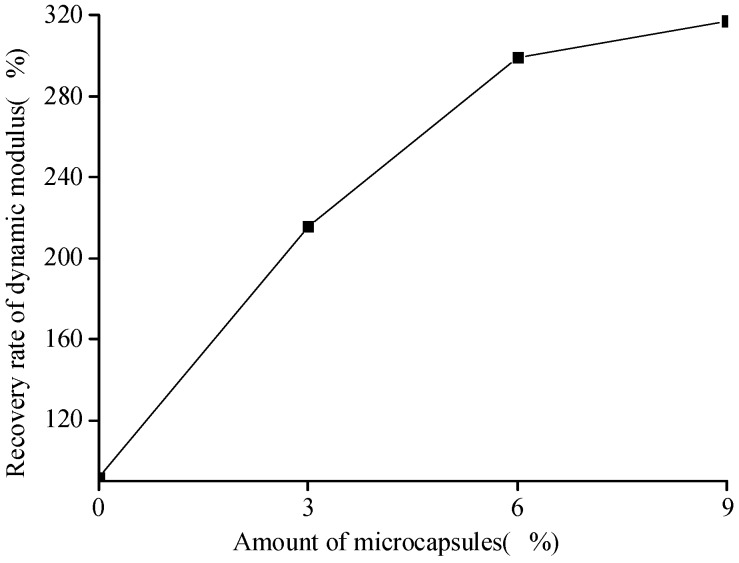
Recovery rate (Equation (5)) of the dynamic modulus with amount of microcapsules.

Figure 8 and Figure 9 show that the healing and recovery rates of the dynamic modulus of the cement-based materials increase with the increase in the amount of microcapsules. The healing and recovery rates of the samples without the microcapsules were 16.95% and 91.30%, respectively. The healing rate was positive, but lower, indicating that the microcracks in the specimen without the microcapsules can achieve self-healing by extending the hydration. However, this effect is limited, as it cannot restore the initial state of the specimens. The healing and recovery rates of the composites with microcapsules were higher than those of the specimens without microcapsules. The results show that the effect of extending the hydration is limited, and the microcapsules have a significant effect on the self-healing process of the cement-based material. It indicates that, the more the amount of microcapsules, the more the healing agent; the self-healing effect of the cement-based material is more evident. It is found that compared to the compressive strength of the specimen, the dynamic modulus has considerably higher healing rate with the microcapsules. However, as the microcapsules decrease the initial value of the elastic modulus and strength, clearly, an appropriate value of the amount of microcapsules should be determined because of the target performance.

### 3.3. MIP Results and Discussions

**Figure 10:**
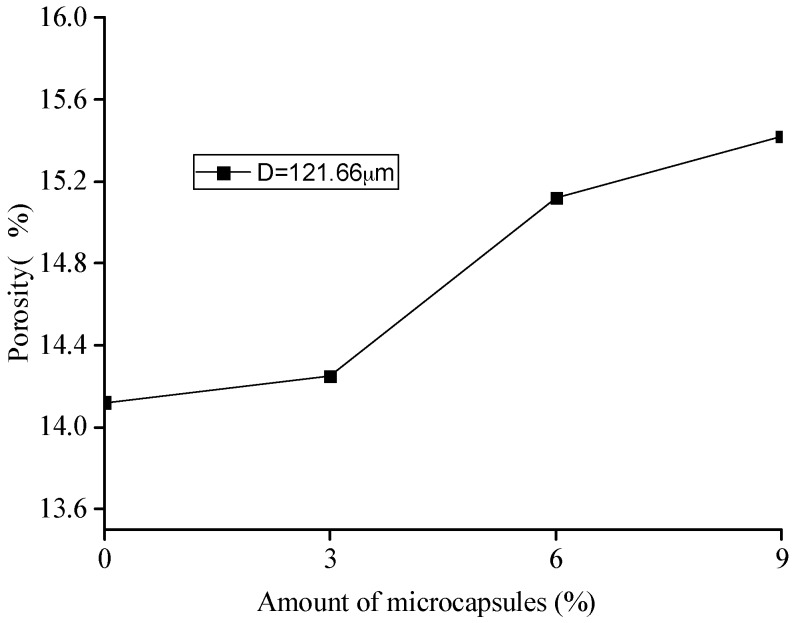
Variation in the porosity with amount of microcapsules.

**Figure 11:**
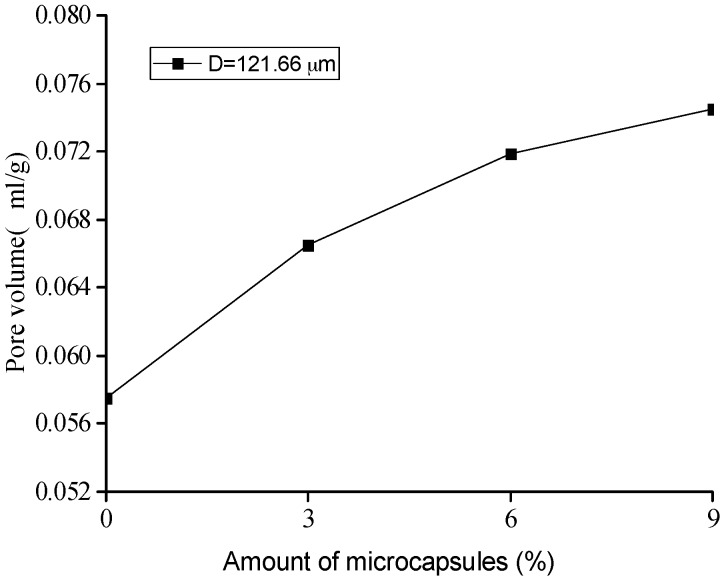
Variation in the pore volume with amount of microcapsules.

**Figure 12:**
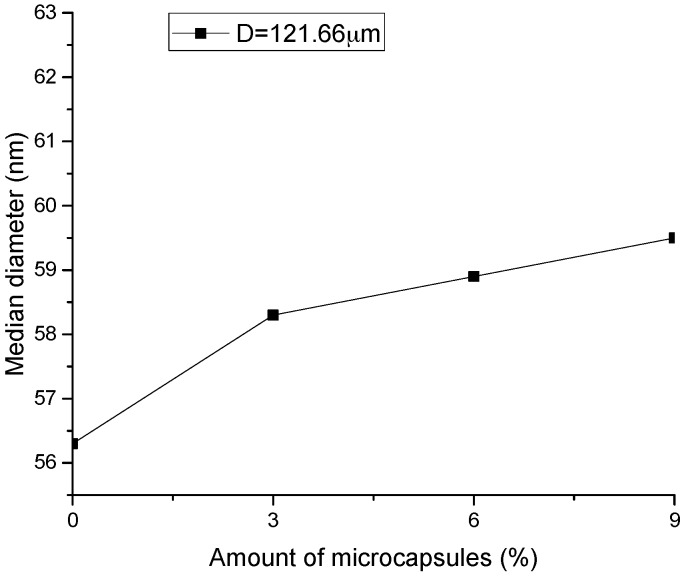
Variation in the median pore diameter microcapsules.

**Figure 13:**
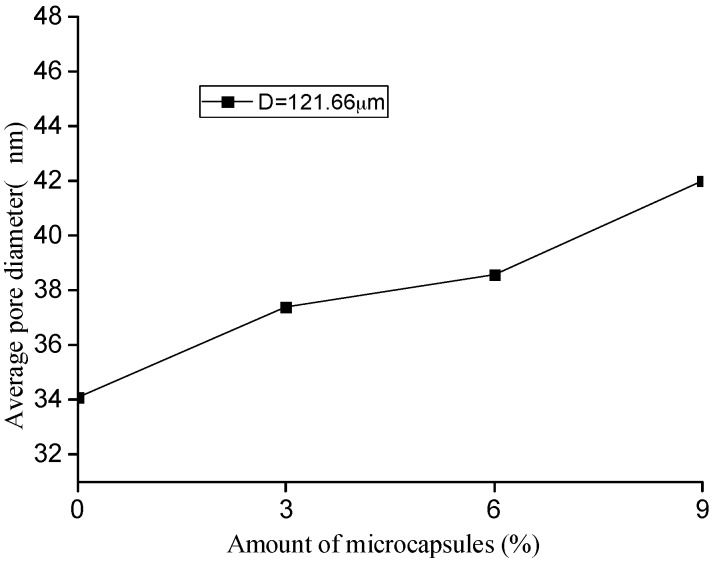
Variation in the average pore diameter with amount of microcapsules.

Based on the MIP test, the pore-structure parameters, such as porosity, pore volume, median value, and the average value of the pore size for the original specimens with different amount of microcapsules are shown in Figure 10, Figure 11, Figure 12 and Figure 13. It is seen that all the values of the porosity, pore volume, median value, and average value of the pore size increase with the increase in the amount of microcapsules (0%, 3%, 6%, and 9%). However, in the case of 3% microcapsules, the porosity of the specimen is almost the same as the one without the microcapsules. This can also explain the filler effect of the microcapsules when the amount of the mixed microcapsules is small. The variation in the total pore volume of the specimen shows an opposite trend compared to the results of the nitrogen adsorption test (Brunauer–Emmett–Teller (BET)) [34]. The volume of the pores less than 50 nm measured by the BET reduced with the amount of microcapsules. However, it increased when the MIP test was employed, indicating that, both the volume of the pores greater than 50 nm and the number of harmful holes increased. The increase in the porosity, average pore diameter, and the critical diameter indicated an increase in the number of large pores. Then, the increase in the amount of microcapsules increases the large pores and further affects the strength and permeability of the specimen.

**Figure 14:**
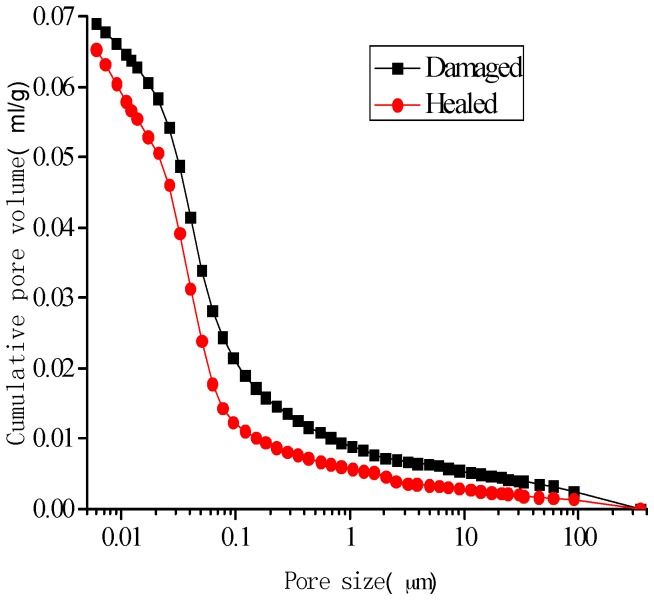
Cumulative-pore volume distribution for specimens without microcapsules.

**Figure 15:**
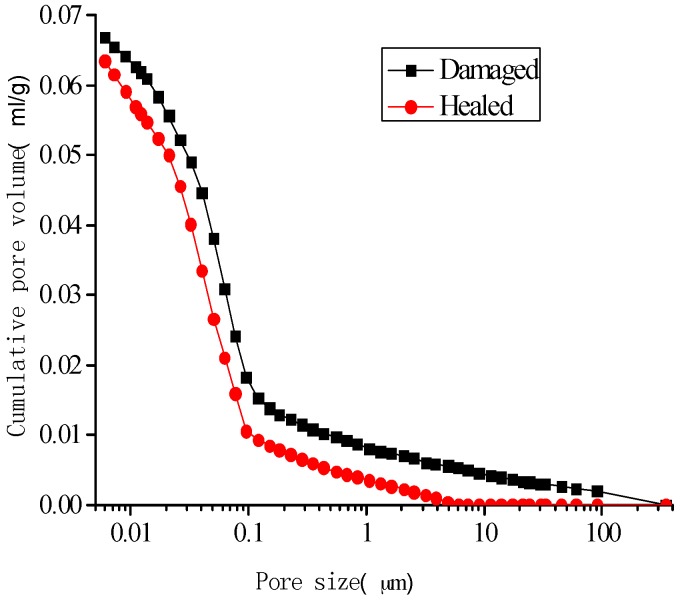
Cumulative-pore volume distribution for specimens with 3% microcapsules.

**Figure 16:**
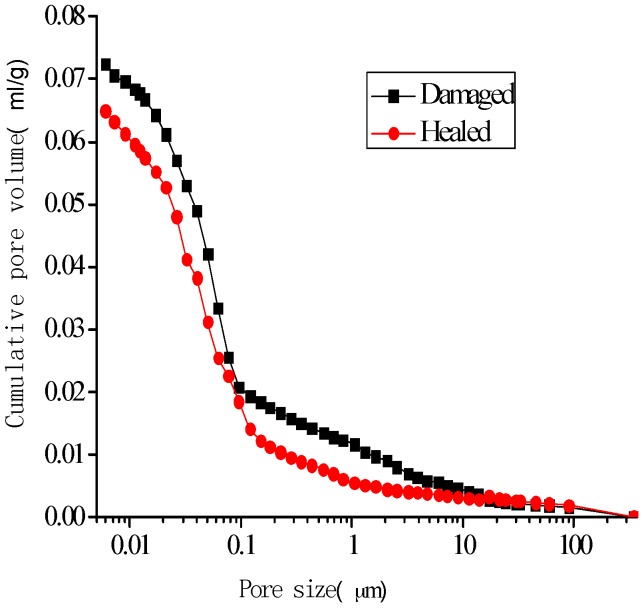
Cumulative-pore volume distribution for specimens with 6% microcapsules.

**Figure 17:**
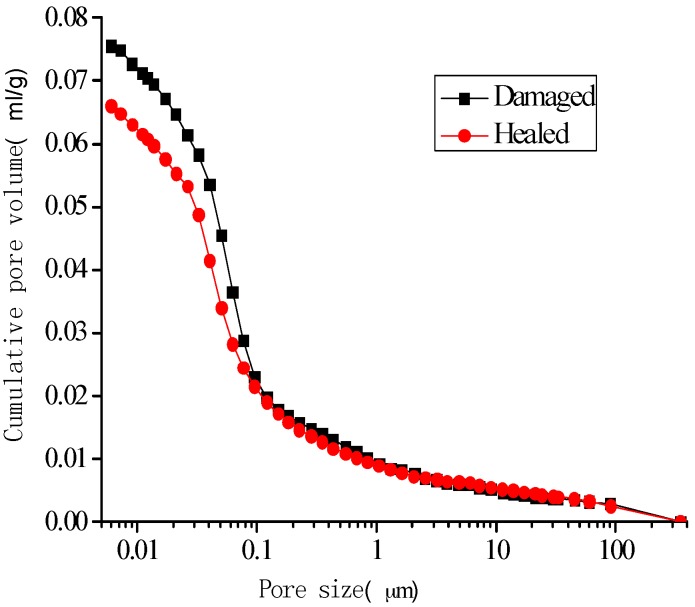
Cumulative-pore volume distribution for specimens with 9% microcapsules.

Figure 14, Figure 15, Figure 16 and Figure 17 show the pore-size distribution of the specimens before and after the self-healing for the microcapsule size of 121.66 μm for the different amount of microcapsules (0%, 3%, 6%, and 9%), respectively. It can be seen that for all the percentages of microcapsules, the distribution of the cumulative-pore volume after the healing showed lower values than those under damage.

To investigate the self-healing behavior at the pore-structure level, a series of definitions for the pore structure parameters are given as

(7) $$\eta_{P}=\frac{P_{damaged}-P_{healed}}{P_{damaged}}\times 100\%\;,\;\eta_{RP}=\frac{P_{original}}{P_{healed}}\;\times 100\%\;,\;$$

(8) $$\eta_{V}=\frac{V_{damaged}-V_{healed}}{V_{damaged}}\times 100\%\;,\;\eta_{RV}=\frac{V_{original}}{V_{healed}}\;\times 100\%\;,\;$$

(9) $$\eta_{AVE}=\frac{A_{damaged}-A_{healed}}{A_{damaged}}\times 100\%\;,\;\eta_{RAVE}=\frac{A_{original}}{A_{healed}}\;\times 100\%\;,\;$$

where $\eta_{P}$, $\eta_{V}$, and $\eta_{AVE}$ denote the healing rates of the porosity, total pore volume, and average pore diameter of the specimen, respectively; $\eta_{RP}$, ${\;\eta }_{RV}$, and $\eta_{RAVE}$ represent the recovery rates of the porosity, total pore volume, and average pore diameter of the specimen, respectively; $P_{original}$, $V_{original}$, and $A_{original}$ are the porosity, total pore volume, and average pore diameter of the original specimen, respectively; $P_{damaged}$, $V_{damaged}$, and $A_{damaged}$ are the porosity, total pore volume, and average pore diameter of the damaged specimen, respectively; $P_{healed}$, $V_{healed}$, and $A_{healed}$ are the porosity, total pore volume, and average pore diameter of the healed specimen, respectively.

**Figure 18:**
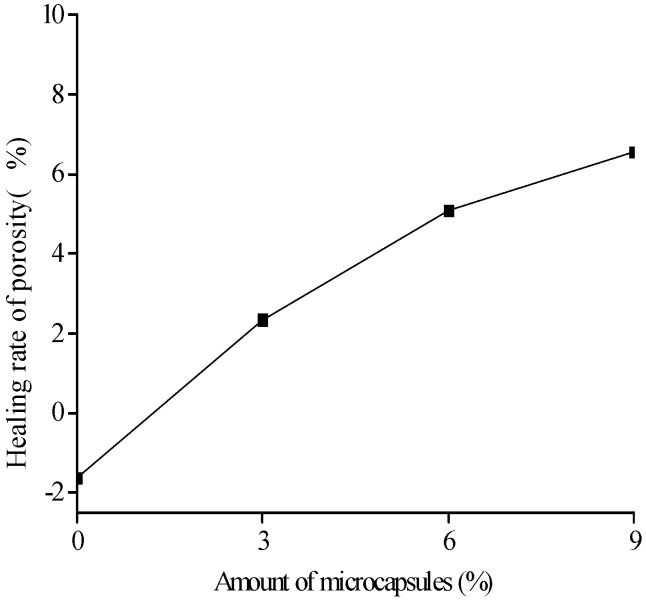
Healing rate (Equation (7): $\eta_{P}$) of porosity with amount of microcapsules.

**Figure 19:**
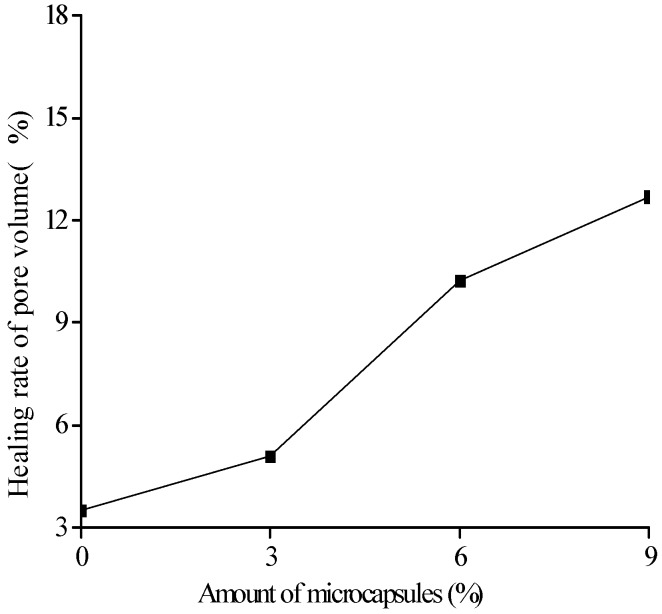
Healing rate (Equation (8): $\eta_{V}$) of pore volume with amount of microcapsules.

**Figure 20:**
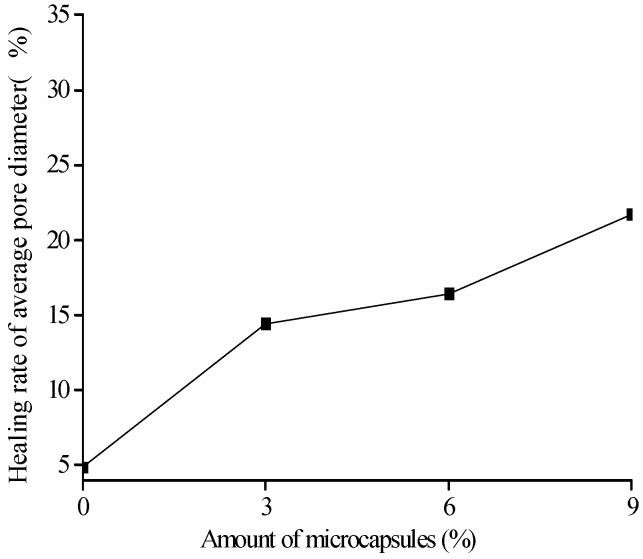
Healing rate (Equation (9): $\eta_{AVE}$) of average pore diameter with amount of microcapsules.

**Figure 21:**
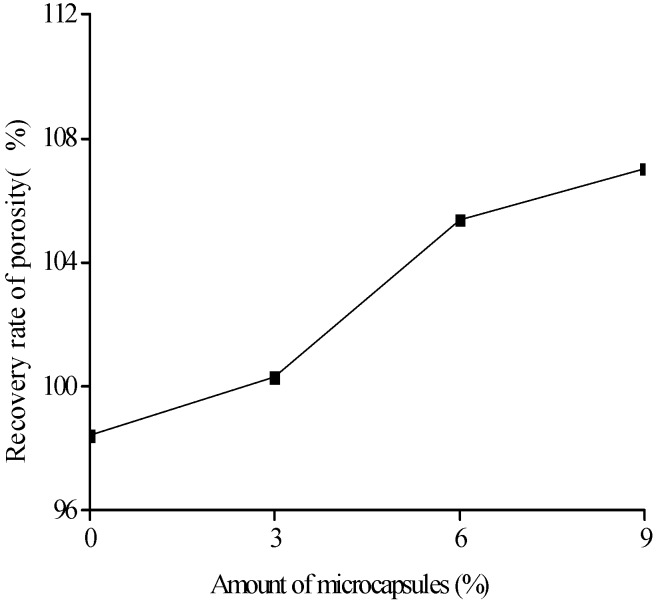
Recovery rate (Equation (7): $\eta_{RP}$) of porosity with amount of microcapsules.

**Figure 22:**
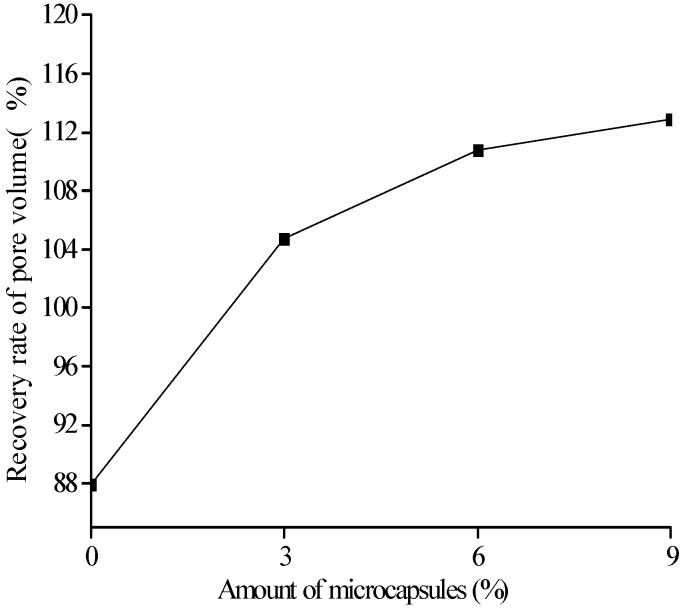
Recovery rate (Equation (8): $\eta_{RV}$) of pore volume with amount of microcapsules.

**Figure 23:**
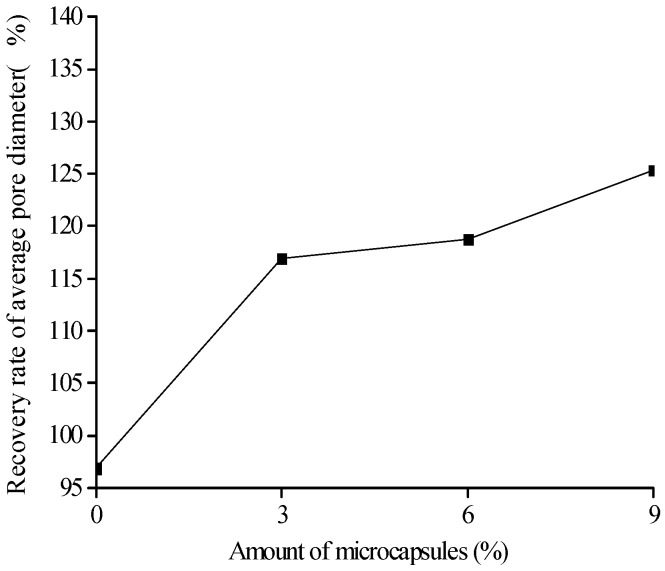
Recovery rate (Equation (9): $\eta_{RAVE}$) of average pore diameter with amount of microcapsules.

Figure 18, Figure 19 and Figure 20 show the healing rates of the porosity, total pore volume, and average pore diameter of the specimen, respectively, with the amount of microcapsules. Figure 21, Figure 22 and Figure 23 show the recovery rates of the porosity, total pore volume, and average pore diameter of the specimen, respectively, with increasing amount of microcapsules. It is noted that the variation in both the healing and recovery rates of the pore structure parameters, such as the porosity, total pore volume, and average pore diameter of the specimen are consistent; they increase with the amount of microcapsules. The results of the compressive strength and dynamic modulus, given in the previous section, shows that the healing and the recovery rates of the pore structure parameters are consistent with the macro mechanical behaviors. It is validated that the microcapsules can provide self-healing function for the cementitious composite; however, it is shown that the pore structure parameters have important influences on the mechanical behaviors such as compressive strength and dynamic modulus.

## 4. Conclusions

The microcapsules-based self-healing cementitious composite was developed in Guangdong Provincial Key Laboratory of Durability for Marine Civil Engineering, Shenzhen University. Both the macro performance, such as the compressive strength and the dynamic modulus, and the microstructure of the pore-structure parameters were investigated. The results reflect the consistency between the macro behavior and the microstructures. It is concluded that, for the original specimen, both the compressive strength and the dynamic modulus, as well as the pore structure parameters, such as porosity, cumulative pore volume, and average pore diameter, decrease to some extent with increasing amount of microcapsules. However, the self-healing and recovery rates of the specimen performance and the pore structure parameters increase with increasing the amount of microcapsules. Therefore, for the future practical application, there is a need to balance the amount of microcapsules in order to achieve the self-healing function and avoid the drawback of the effect of the microcapsules themselves.

## Figures and Tables

**Table 1:** Microcapsules parameters.

Rotation Velocity in Synthesis (r/min)	Mean Diameter (μm)	Wall Thickness (μm)	Capsule Core Content (%)
600	121.66	5.46	67.8

**Table 2:** Mix proportions of specimens.

Sample No.	1	2	3	4
Particle diameter (μm)	N/A	121.66	121.66	121.66
Microcapsule content (to cement mass)	0%	3%	6%	9%

**Table 3:** Test No. and types.

	Sample No.	1	2	3	4	Test Type
No.						
1		1-1-1	2-1-1	3-1-1	4-1-1	Compression test,
2		1-1-2	2-1-2	3-1-2	4-1-2	DMA, MIP test
3		1-2-1	2-2-1	3-2-1	4-2-1	DMA test
4		1-2-2	2-2-2	3-2-2	4-2-2	MIP test to damaged sample
5		1-3-1	2-3-1	3-3-1	4-3-1	Compression test to healed sample
6		1-3-2	2-3-2	3-3-2	4-3-2	DMA, MIP test
